# Early MCI-to-AD Conversion Prediction Using Future Value Forecasting of Multimodal Features

**DOI:** 10.1155/2021/6628036

**Published:** 2021-09-24

**Authors:** Sidra Minhas, Aasia Khanum, Atif Alvi, Farhan Riaz, Shoab A. Khan, Fawaz Alsolami, Muazzam A Khan

**Affiliations:** ^1^Department of Computer Science, Forman Christian College University, Lahore, Pakistan; ^2^Department of Computer Science, University of Management and Technology, Lahore, Pakistan; ^3^Department of Computer Engineering, National University of Sciences & Technology, EME College, Rawalpindi, Pakistan; ^4^Department of Computer Science, King Abdulaziz University, Jeddah, Saudi Arabia; ^5^Department of Computer Sciences, Quaid I Azam University, Islamabad, Pakistan

## Abstract

In Alzheimer's disease (AD) progression, it is imperative to identify the subjects with mild cognitive impairment before clinical symptoms of AD appear. This work proposes a technique for decision support in identifying subjects who will show transition from mild cognitive impairment (MCI) to Alzheimer's disease (AD) in the future. We used robust predictors from multivariate MRI-derived biomarkers and neuropsychological measures and tracked their longitudinal trajectories to predict signs of AD in the MCI population. Assuming piecewise linear progression of the disease, we designed a novel weighted gradient offset-based technique to forecast the future marker value using readings from at least two previous follow-up visits. Later, the complete predictor trajectories are used as features for a standard support vector machine classifier to identify MCI-to-AD progressors amongst the MCI patients enrolled in the Alzheimer's disease neuroimaging initiative (ADNI) cohort. We explored the performance of both unimodal and multimodal models in a 5-fold cross-validation setup. The proposed technique resulted in a high classification AUC of 91.2% and 95.7% for 6-month- and 1-year-ahead AD prediction, respectively, using multimodal markers. In the end, we discuss the efficacy of MRI markers as compared to NM for MCI-to-AD conversion prediction.

## 1. Introduction

Alzheimer's disease (AD) is currently the most prevalent and deadliest form of dementia amongst the elderly—its fatality has increased by 68%, while other major disorders have decreased (Alzheimer's Association 2014). Several serious attempts have been made at disease staging and early diagnosis over the past half-century to curb this high rate. In 2011, the National Institute of Ageing and Alzheimer's Association presented revised guidelines for diagnosing AD [1]. They divided the disease into three stages: (1) preclinical AD, in which measurable changes in biological and pathological markers occur; however, no outward changes are observed, (2) mild cognitive impairment (MCI), during which slight memory and cognitive complaints are observed and measured, and (3) dementia due to AD, when the patient cannot perform daily life activities due to memory and cognition problems. It is also established that not all MCI patients necessarily develop AD in the future [2]. Generally, there are two kinds of clinical changes for MCI patients: (1) MCI stables (MCIs) are those who retain MCI diagnosis at future time points and (2) MCI progressors (MCIp) are those who show symptoms of AD in the future. Hence, it is imperative to develop reliable techniques to foresee the future clinical trends in MCI patients which can later be used to categorize them as MCIp or MCIs.

A variety of predictors have been proposed for early diagnosis of AD ranging from noninvasive neuropsychological measures (NM) [3] and structural magnetic resonance imaging (MRI) to others like positron emission tomography (PET), chemical content of cerebrospinal fluid (CSF), and genetic sampling [4]. Recent research is focused on the efficacy of these markers for detecting MCI progression to AD. Numerous studies examined the role of cognitive markers for the conversion prediction task [5–7], while various other projects resorted to scrutinizing the biological markers [8–11]. Similarly, a plethora of studies reported enhanced predictive power of strategies involving multimodal information [12–16], while many authors proposed composite predictors of the latent disease [17, 18]. Among several studies, the authors of [9, 19] presented effective feature selection strategies incorporating regularized linear regression and deep neural networks, respectively. However, a presentation by Sperling et al. [1] indicates that cognitive function and brain structure deteriorate more sharply during MCI to AD transition than other factors like protein deposition. Similarly, NM and MRI measures are most prevalent in the literature as they are comparatively cheaper and easier to obtain than CSF measures which require lumbar puncture. Most of these studies resort to using the baseline data only, whereas, in practice, data from multiple time points may contain helpful information regarding the disease's underlying pathology.

Identification of AD as a slowly progressing disease has gained interest of many researchers. AD must be monitored over time to capture the predictor variability effectively and understand the disease progression. Hence, to predict MCI conversion to AD, the researchers started taking into account the longitudinal data instead of one time-point data [8, 9, 20, 21]. Huang et al. [22] quantified the 6-month-apart longitudinal change observed in significant voxels of MRI images using linear regression and, later, employed a hierarchical classifier to categorize MCIp and MCIs. Their longitudinal method achieved a classification accuracy of 79.4% compared to 71.7% accuracy obtained when they used baseline data only, hence reinforcing the abilities of longitudinal data. Lee et al. [23] investigated the effects of longitudinal callosal atrophy to predict MCI to AD conversion. Their method was found to be more accurate (84%) for female patients than male patients (61%). Another powerful method for MCI progression was presented by Runtti et al. [24], who employed neuropsychological measures and heterogeneous biomarkers to calculate the Disease State Index (DSI) of all patients. Previous DSI measures were then used to estimate the next DSI using linear regression. The classification accuracy achieved by this method was 76.9%. Guo et al. [25] proposed a robust, novel method using three-year follow-up data to mine a dynamic MRI morphological feature and based MCI-to-AD conversion prediction on it. The classification accuracy obtained by Guo et al. was 84.62%. A few other significant studies employing longitudinal predictor information are summarized in Table 1. Lawrence et al. [28], apart from advocating the superior power of time-series data for early AD prediction, provided a detailed review of studies to gather longitudinal data for this domain. However, the researchers are still faced with the sparsity of data on CSF and PET scans for the said task.

A variety of machine learning and pattern recognition techniques have been validated and tested in the MCI-to-AD conversion prediction domain, which includes supervised [9, 16], unsupervised, and semisupervised [11, 29] methods. Further stratifications involve classification and regression [15, 21] techniques. While SVM and its variants have been a classical choice for early diagnosis of AD [26], several researchers opted to use random forests [11], hierarchical classifiers [22], and maximum uncertainty linear discriminant analysis [30]. Despite many efforts, the desired performance in terms of AUC and accuracy for a stable clinical decision support technique has not been achieved. In many studies, a tradeoff between sensitivity and specificity of the system is observed. From most of the earlier studies, it is extracted that the quality of predictors is more crucial in detecting MCI progression than the classifier's sophistication. Recognition of an effective feature set for classification is essential. Another noticeable fact that resulted in low performance of systems for early diagnosis of AD is the use of one time-point data only. Peterson et al. [31] reinforce that the rates of clinical changes in MCI subjects are meaningfully rapid than the controls, hence enhancing our interest in quantifying those changes.

In this paper, we propose a multistep method to forecast next-in-sequence predictor readings engaging the previous two readings and later use the time-series information for MCIp and MCIs classification employing an SVM classifier. Our experiments incorporate nonbrain neuropsychological measures (NM) and brain MRI-derived measures as predictors of MCI progression to AD. We designed a novel weighted gradient offset method, which calculates the future predictor reading employing two consecutive, previously available, time-domain readings of multimodal predictors. The future value forecasting system is governed by quantifying previous gradient changes onto the next interval. The novelty of the proposed method lies in the effective employment of several bimodal predictors to forecast future predictor readings. We validate the proposed method on Alzheimer's Disease Neuroimaging Initiative (ADNI) data set. The efficacy of our system is judged on nonbrain and brain predictors in individual and combined settings.

## 2. Materials

### 2.1. ADNI

Data used in this work are obtained from Alzheimer's Disease Neuroimaging Initiative (ADNI) database. ADNI is a large-scale multisite study that aims at analyzing markers from cognitive tests, blood tests, tests of CSF, and MRI/PET imaging concerning their ability to characterize the progression of Alzheimer's disease. ADNI results from the efforts of many co-investigators from a broad range of academic institutions and private corporations. Subjects were recruited from over 50 sites across the US and Canada. ADNI data used in this study were retrieved in January 2019. Till then, ADNI, in its three studies (ADNI-1, -GO, and -2), had recruited over 1500 adults aged between 55 and 90 years to participate in this study. For up-to-date information, see http://www.adni-info.org.

Here, for technique validation, we consider the subset of MCI subjects enrolled in ADNI-1 with at least three consecutive follow-up readings available. The subjects with MCI converting to AD any time before the last follow-up time, i.e., 36^th^ month, are called MCI progressors (MCIp), and those retaining MCI diagnosis at all times are known as MCI stables (MCIs). Subjects with missing values at designated follow-up times are ignored. For 6-month-ahead prediction, the dataset consists of subjects with three consecutive 6 monthly follow-up readings. Therefore, we have 49 MCIp and 70 MCIs subjects whose IDs are given in the supplementary files (available here). Similarly, for 1-year-ahead prediction, the dataset contains three consecutive annual readings, resulting in 35 MCIp and 50 MCIs subjects. Subject demographic information at baseline is stated in Table 2.

### 2.2. Markers

A study conducted by Sperling et al. [1] demonstrates that in contrast to chemical and protein compositions, brain morphometry, clinical function, and cognitive performance are most affected during MCI-to-AD transition. Hence, in this work, we concentrate on longitudinal analysis and use of nonbrain neuropsychological markers (NM) and brain MRI morphometric markers only. Further details about the markers considered in this study are specified as follows.

#### 2.2.1. Neuropsychological Markers (NM)

ADNI participants are asked to perform a series of neuropsychological tests and clinical assessments regularly. The scores and values of each test and assessment are recorded. The NM employed in this work are identified in [32]. In [33], Pereira et al. comprehensively studied the impact of various neuropsychological measures on MCI-to-AD conversion prediction, and based on those findings, we select a total of eight markers which are as follows: total scores of AD Assessment Scale (ADAS 13), Rey Auditory Verbal Test (RAVLT), Clock Drawing Test (CDT), Clock Copying Test (CCT), Immediate Recall Total Score (LIMM), Mini-Mental State Examination (MMSE), Trail Making Test A (TRAA), and Trail Making Test B (TRAB).  Table 3 describes the mean and standard deviations observed in these values. The test procedures and scoring criteria for the selected cognitive tests are given in the ADNI General Procedures Manual [32].

#### 2.2.2. MRI Morphometric Markers

For MCI-to-AD conversion prediction, we also employ the longitudinal MRI morphometric measures provided by the University of California, San Francisco, Memory and Ageing Centre, on the ADNI website. The complete details of extracting the MRI-derived measures are provided in [32]. Briefly, T1-weighted, MPRAGE MR scans from a 1.5 T Siemens scanner (dimensions 1 mm × 1 mm x 1 mm, TR: 20 ms, TE: 5 ms) were downloaded and preprocessed for gradient warping, scaling, B1 correction, and N3 inhomogeneity correction by Mayo Clinic [34–36]. Cortical reconstruction and volumetric segmentation were then performed using Freesurfur image analysis suite version 4.3 based on the framework provided in [37]. In the said framework, a within-subject template space and average image, unbiased towards the chronological scan order, was created using robust, inverse consistent registration [38, 39]. Cortical morphometric measures were obtained after iterative topology correction, nonlinear Atlas registration, and nonlinear spherical surface registration. The segmented images were passed through an intensive quality control (QC) process [40] by Mayo clinic and provided for use on the ADNI website. The current study includes the information only from those images which passed the overall QC process. The MRI biomarkers consist of volumes of brain regions obtained after cortical parcellation and white matter parcellation, the surface area of the brain regions, and cortical thickness of the brain regions provided in the UCSFFSL file on the ADNI website. We consider a total of 249 brain MRI features in the current study.

## 3. Methods

**Figure 1:**
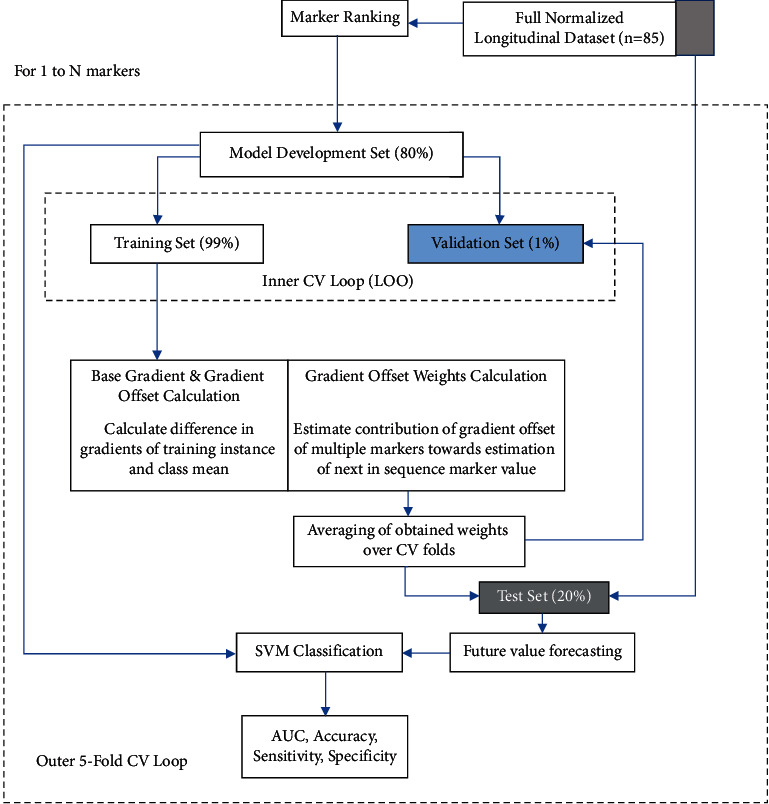
System overview.

Our work focuses on utilizing the embedded predictive power in longitudinal features for MCI progression prediction. We use multimodal (nonbrain NM and brain MRI) markers recorded at two consecutive follow-ups for MCI subjects and pass them through a pipeline to forecast the next marker value. The time-domain readings (three in number) are then collectively considered for MCIp or MCIs label prediction. The detailed process overview is shown in Figure 1, which shows nested cross-validation loops with a 5-fold outer loop for classification and an inner leave-one-out loop for average weights calculation. The process is repeated for all feature subsets.

The proposed approach for longitudinal trajectory modeling is three-phased: (1) estimating gradient offset and its weights from the training data, (2) forecasting future marker values for the test data using models generated by the training data, and (3) classifying the time-domain trajectories of test instances as one of the two classes. The system is implemented on MATLAB R2018a. Details of each module are in the following sections.

### 3.1. Preprocessing

#### 3.1.1. Marker Normalization

Feature normalization is an essential step in a multivariate, multiscaled machine learning environment. Normalization is performed to remove bias due to very small or very large-scaled values. We scale all features to have values between 0 and 1 before the train-test split. For longitudinal dataset normalization, we divide the time-domain trajectories of each biomarker by the maximum value of the respective biomarker to produce values between 0 and 1 while preserving the longitudinal trends.

#### 3.1.2. Marker Ranking and Selection

The biomarker set we consider in this work consists of 249 MRI and 8 NM biomarkers, of which not all contribute effectively towards MCIp vs. MCIs segregation. For optimal biomarker subset determination, we adopt a wrapper-based approach for selecting significant biomarkers. We perform two sampled Student's *T*-tests on baseline biomarker readings and then sift them according to their ranks in impact towards MCIp vs. MCIs discrimination. The *p*  values of the *T*-test indicate the significance of a particular biomarker towards effective diagnostics. Later, we form biomarker subsets by incrementally adding one biomarker at a time according to its rank. Each biomarker subset is modeled and evaluated for its efficacy through our proposed system.

### 3.2. Longitudinal Trajectory Modeling

To forecast future clinical changes of an MCI subject, i.e., at *t*+2Δ*t*, a reliable predictor model must capture the effects of past feature readings (at *t* and *t*+Δ*t*) and project them onto the future clinical readings. Assuming longitudinal trajectories to be piecewise, we select a linear progression model for MCI-to-AD progression to avoid overfitting to a small dataset [41, 42].

For weights calculation and model validation, we divide the dataset into a 5-fold cross-validation setup. The training set is further stratified into training and validation set according to the leave-one-out (LOO) scheme for hyperparameter tuning. Let *X*_*c*_={*X*_1_, *X*_2_,…, *X*_*N*_} be the training set of all *N* longitudinal predictors of *n* MCI subjects belonging to class *c* (*c* = 1 for MCIp and *c* = 0 for MCIs). Then, $X_{m}=\left[\begin{array}{ccc} x_{mi}\left(t\right) & x_{mi}\left(t+\Delta t\right) & x_{mi}\left(t+2\Delta t\right) \end{array}\right],\text{ for }m=1,\ldots ,N,i=1,\ldots ,n,\Delta t=0.5,1$, where Δ*t* is 0.5 for 6-month-ahead prediction and 1 for one-year-ahead prediction. The base gradient, *δ*_*m*_^*c*^(Δ*t*), quantifies the average piecewise change for the *m*^th^ predictor's trajectory belonging to class *c.* To obtain *δ*_*m*_^*c*^(Δ*t*), we subtract *x*_*mi*_(*t*) from *x*_*mi*_(*t*+Δ*t*), divide it by Δ*t*, and average it over the total number of instances in the respective class.

Similarly, we calculate the piecewise gradient between consecutive longitudinal values of the validation instance, *δ*_*mi*_(Δ*t*), using Euclidean geometry as *δ*_*mi*_(Δ*t*)=*x*_*mi*_(*t*+Δ*t*) − (*x*_*mi*_(*t*)/Δ*t*) for *m*=1,…, *N*, *i*=1,…, *n*. The validation subject's piecewise gradient, *δ*_*mi*_(Δ*t*), is subtracted from the respective base gradient *δ*_*m*_^*c*^(Δ*t*) to obtain the gradient offsets OS_*mi*_(Δ*t*).

**Figure 2:**
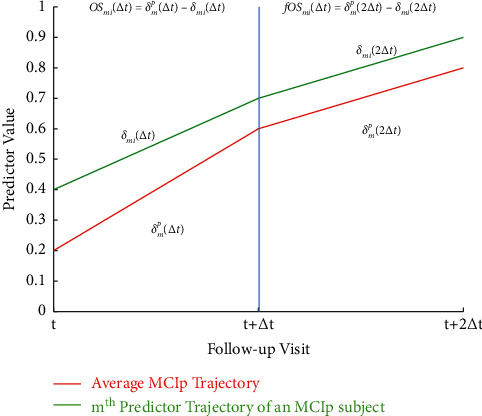
Gradient offset calculation.

We aim to measure the extent to which the gradient offsets between interval *t* and *t*+Δ*t*, i.e., OS_*mi*_(Δ*t*), affect the future gradient offset fOS_*mi*_(Δ*t*), over the interval *t*+Δ*t* and *t*+2Δ*t* of a particular marker *m.* The process of obtaining gradient offsets is graphically depicted in Figure 2. We model the gradient offset relationship between the current and next follow-up interval by an over-determined system of linear equations modulated by the vector of linear prediction coefficients *μ*. For a particular marker *m*, this relationship is represented by

(1) $$\begin{array}{c} A\times \mu =B_{m},\;\text{where }A=\left[\begin{array}{ccc} {\text{OS}}_{11}\left(\Delta t\right) & \cdots & {\text{OS}}_{N1}\left(\Delta t\right)\\ \vdots & \ddots & \vdots \\ {\text{OS}}_{1n}\left(\Delta t\right) & \ldots & {\text{OS}}_{Nn}\left(\Delta t\right) \end{array}\right],\\ \mu =\left[\begin{array}{c} \mu_{11}\\ \vdots \\ \mu_{Nn} \end{array}\right]\text{ and }B=\left[\begin{array}{c} {\text{fOS}}_{m1}\left(\Delta t\right)\\ \vdots \\ {\text{fOS}}_{mn}\left(\Delta t\right) \end{array}\right],\text{ for }m=1,\ldots ,N. \end{array}$$

We solve equation (1) for *µ* such that the squared error between predicted and actual offsets is minimized. The derivative of the squared error is equated to 0, and the final formulation of *μ* is obtained by *µ*=(**A**^**T**^**A**)^−1^**A**^**T**^**B**_**m**_. *μ* is the vector containing the values of linear prediction weights quantifying the effects of heterogeneous, multivariate markers on the future values of the marker under consideration. For an optimal model generation, we average the obtained weights over LOO validation folds.

### 3.3. Future Value Forecasting

In this work, we forecast the future clinical changes of an MCI subject using the previous two consecutive follow-up readings of heterogeneous markers to enhance classification performance for MCIp vs. MCIs. Let *y* be a test instance with two known predictor readings for a predictor *m* as *y*_*m*_(*t*) and *y*_*m*_(*t*+Δ*t*) and piecewise linear relation between them. We calculate the future values, *y*_*m*_(*t*+2Δ*t*), using Euclidean geometry according to

(2) $$\begin{array}{c} y_{m}\left(t+2\Delta t\right)=y_{m}\left(t+\Delta t\right)+\omega_{m}*t,\;\text{where }\omega_{m}=\alpha_{m}+\beta_{m}\text{ for }m=1,\ldots ,N. \end{array}$$

Here, *ω*_*m*_ is the piecewise gradient between (*t*+Δ*t*) and (*t*+2Δ*t*) of a marker *m*. We calculate *ω*_*m*_ by adjusting the weighted gradient offset *β*_*m*_ to the base gradient *α*_*m*_. The methods to obtain *α*_*m*_ and *β*_*m*_ are detailed in the following.

#### 3.3.1. Base Gradient *α*_*m*_

We evaluate the points *y*_*m*_(*t*) and *y*_*m*_(*t*+Δ*t*) for their belongingness to one of the groups, MCIp or MCIs, using the known time-point marker values and annual change in those values as features. These features are fed to a linear support vector machine (SVM) classifier. SVM is selected for binary classification based on limited dataset availability and the robustness of the said classifier [43]. Upon the suggestion of the base group by the SVM classifier, we substitute the average annual change, *δ*_*m*_^*c*^(Δ*t*), of the respective class for *α*_*m*_.

#### 3.3.2. Weighted Gradient Offset *β*_*m*_

It is important to incorporate the effects of multivariate markers in forecasting future marker readings. In this study, we integrate the multivariate effects into the gradient offset measure which is added in *α*_*m*_. The gradient between the recorded follow-up readings *δ*_*y*_*m*__(Δ*t*) of *y*_m_ is calculated by subtracting *y*_*m*_(*t*) from *y*_*m*_(*t*+Δ*t*) and dividing it by Δ*t*. Gradient offset for the marker OS_*y*_*m*__ is calculated by OS_*y*_*m*__=*α*_*m*_ − *δ*_*y*_*m*__, which quantifies the difference between the base gradient and the actual predictor gradient.

To incorporate the effects of multiple predictors onto the future value of the predictor under consideration, we multiply the vector of linear prediction weights *µ* by the offset vector OS_*y*_*m*__ to obtain *β*_*m*_ according to

(3) $$\begin{array}{c} \beta_{m}=\;\mu \times {\text{OS}}_{y_{m}}^{T},\;\text{for }m=1,\ldots ,N. \end{array}$$

We substitute the obtained values of *α*_*m*_ and *β*_*m*_ in equation (2) to forecast the following value of the predictor *m* of the test MCI subject. This method is repeated for all *N* markers being considered in the marker subset to obtain complete time-domain trajectories for the multivariate markers.

### 3.4. Trajectory Classification

Once the complete time-point biomarker trajectories for individual MCI patients are obtained, we classify them as MCIp and MCIs using a standard linear SVM classifier. After classification, we assign a class label and likelihood values for the respective class to each MCI individual.

### 3.5. Performance Evaluation

To analyze the effectiveness of future biomarker values forecasting, we record the mean absolute error (MAE) between the predicted and observed values. Later, to quantify the performance of MCIp vs. MCIs classification, we document the area under ROC (AUC), accuracy, sensitivity, and specificity of the results.

## 4. Results and Discussion

To fully assess the efficiency of our methods, we design three different types of experiments using the following data:

- NM only
- MRI markers only
- NM and MRI combined

For in-depth analysis, we calculate the ground truth (GT) performance scores also in which actual longitudinal marker values, as recorded by ADNI, are used to classify MCIp vs. MCIs, thus overriding the future value-forecasting step. Hence, the GT performance metrics can be used as the benchmark metrics. The aggregate observations from these experiments are described in the following.

### 4.1. Observations about Marker Ranking

Table 4 lists the top five features noted at 6-month- and 1-year-ahead of conversion predictions. The two NM RAVLT and ADAS display the lowest *p*  values, indicating their valuable significance in MCI-to-AD conversion prediction in both long and short periods. We further note that brain volumes are more significant when performing short-term predictions, whereas average cortical thickness proves to be more significant for 1-year-ahead predictions.

### 4.2. Observations about Future Value Forecasting

**Figure 3:**
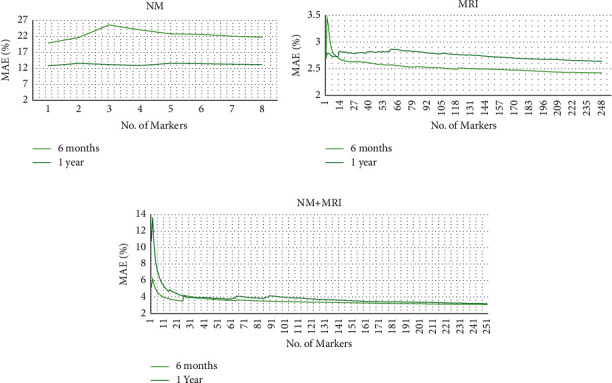
Mean absolute error (MAE) versus number of predictors in the predictor subset for 6-month- and 1-year-ahead future value forecasting using (a) NM only, (b) MRI only, and (c) NM and MRI combined.

The primary objective of this paper is to accurately forecast the marker readings at a future time point based on preceding follow-up data to aid in better MCI-to-AD conversion prediction. We expect that accurate forecasting of a marker reading will decrease the difference between the GT and the proposed system's classification performance. Mean absolute error (MAE) is the metric used to quantify the error between actual and predicted marker values. In Figure 3, we present the trends observed in MAE with the increasing number of predictors for 6-month- and 1-year-ahead value forecasting. In all of the experiments designed (NM, MRI, and NM + MRI), we observe that initially, the forecasting errors increase and are discontinuous but decrease and stabilize later as the number of predictors crosses a certain threshold. Of 257, 98 features present a *p* value of less than 0.05, indicating that the addition of significant markers in the feature set for future value forecasting will enhance the system's classification performance. The minimal effect of low-significance features on MAE when the feature set crosses a certain number of predictors is visible through the tailing of the MAE graphs.

In Figure 3(a), when NM alone is considered for forecasting future time-point readings, the forecasting error reaches the highest (MAE > 12%) amongst all experiments. It reinforces that human behavior is unpredictable, and modeling it can be erroneous, especially over a short (6 months) follow-up duration. On the contrary, in Figure 3(b), experiments considering MRI measures alone report the lowest MAE (<4%) values. The low MAE in MRI measure alone highlights the linear predictability and irreversibility of brain atrophy. 6-month-ahead prediction of MRI markers is more accurate than 1-year-ahead prediction as brain atrophy is a relatively slow process, and minor change occurs between a closer follow-up duration than a longer follow-up duration. In Figure 3(c), we show that the error in forecasting NMs can be reduced by leveraging the power of more predictable MRI measures. The proposed gradient offset method for future value forecasting positively aids in predicting the future course of a marker by employing differential information from multimodal features.

**Figure 4:**
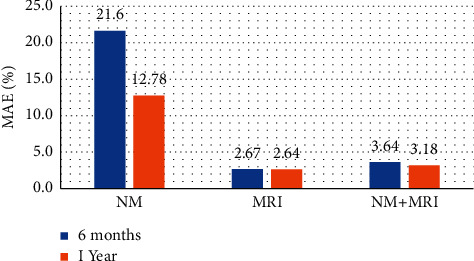
Mean absolute error (MAE) recorded for best performing models in experimental setups for 6-month- and 1-year-ahead marker forecasting.

In Figure 4, we present a comparative summary of MAE of forecasted measures against the classification model, delivering the highest classification AUC. For 6-month-ahead prediction, the NM classification model with 2 markers results in MAE of 21.6% (*p*  value 2.15 × 10^−5^). The MRI classification model with 13 markers presents the lowest MAE of 2.67% (*p*  value 0.0001), while the bimodal setup delivers MAE of 3.64% (*p*  value 0.001) with 19 markers. On the other hand, for 1-year-ahead conversion prediction, we record an MAE of 12.8% (*p*  value 0.0001) using the NM classification model with 4 features, while the best MRI model displays an MAE of 2.6% (*p*  value 0.001) with 13 features. Likewise, the bimodal model with 9 biomarkers delivers an MAE of 3.2% (*p*  value 0.002). We observe lower forecasting errors over a comparatively longer follow-up duration, hence strengthening the fact that longer follow-up durations enhance the predictability of a marker.

### 4.3. Observations about MCIp vs. MCIs Classification Using Longitudinal Data

**Figure 5:**
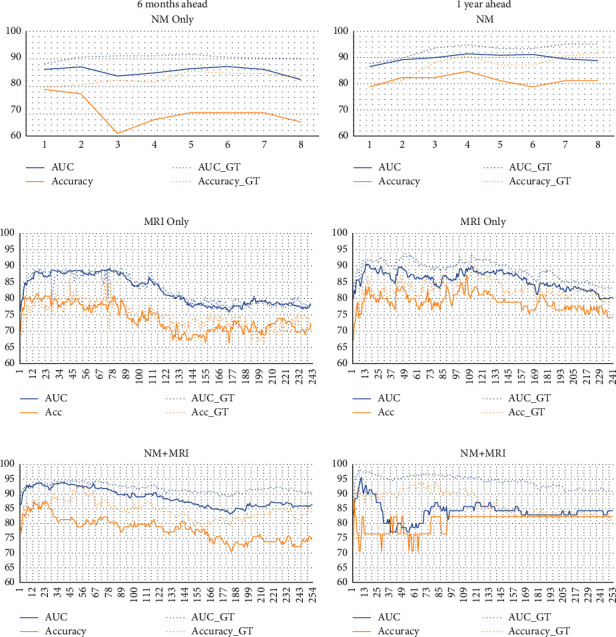
Trends noted in GT and obtained AUC and accuracy for 6-month-ahead (Column 1) and 1-year-ahead (Column 2) conversion prediction.

**Figure 6:**
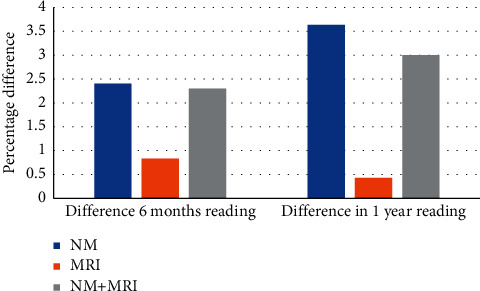
Difference in ground truth and obtained classification AUC for 6-month- and 1-year-ahead conversion prediction.

Figure 5 plots the trends in average AUC (blue lines) and accuracy (yellow lines) metrics of both GT (dotted lines) and proposed (solid lines) classification method for 6-month-ahead (first column) and 1-year-ahead (second column) prediction. From all graphs of Figure 5, we can collectively note that initially, classification performance augments as the number of predictors increases but later, we observe no significant change due to the addition of less-significant features. An interesting observation is made that MRI measures, despite being accurately forecasted, deliver the least classification AUC. This observation is as per previous reports by Brooks et al., which state that longitudinal biological markers are less efficient in capturing AD dynamics than cognitive scores. The same was concluded by Gomar et al. [5, 6] and Ewers et al. [44]. The hypothetical predictor model presented by Sperling et al. [1] also depicts a sharper decline in cognitive performance as compared to structural atrophy of the brain. However, the classification power of our system is the highest when NM measures are used in conjunction with MRI measures which is mentioned in Table 5. In 6-month- and 1-year-ahead predictions, the proposed method delivers a maximum AUC of 91.2% (*p*  value 4.7 × 10^−6^) and 95.7% (*p*  value 4.1 × 10^−7^) using 19 and 9 bimodal features, respectively. In Figure 6, we display the difference between GT AUC and AUC obtained through our system, which is maximum in the case of NM and least in the case of MRI measures. This observation stems from the trends noted earlier that NM are forecasted with the highest error while MRI measures are more accurately forecasted.  Table 6 entails the accuracy, sensitivity, and specificity of the proposed method using the optimal model for both 6-month- and 1-year-ahead prediction. The bimodal setup delivers improved sensitivity and specificity as compared to unimodal models.

The above-stated performance metrics conclude that longer follow-up time and multimodal predictors are favorable for future value forecasting and predicting early AD onset. A combination of biomarkers and cognitive scores augment the confidence in the progressive nature of the underlying disorder. ADAS and RAVLT prove to be the two most informative markers for the said conversion task. These findings have been advocated by many others, including [9, 14, 15, 44].

Table 7 shows a brief comparison of current results with state-of-the-art results obtained on the longitudinal ADNI dataset mainly using NM and MRI data. AUC is considered for comparison to account for imbalance in the data. Misra et al. [8] and Davatzikos et al. [18] employed MRI images to assign abnormality scores to MCIp and MCIs patients. High-dimensional classification methods were applied to the scores to detect MCI progression to AD. They presented respective AUCs of 77% and 73.4% only.

Similarly, Hinrichs et al. [45] proposed a multimodal disease marker using various modalities of data (MRI, PET, and cognitive scores) coupled with SVM classification to identify MCIp patients. The experiments revealed that the proposed technique using longitudinal MRI data produced a maximum MCIp classification AUC of 79%. Moradi et al. [11] also promoted a framework for an aggregate biomarker for MCI conversion prediction using MR imaging data and cognitive scores adjusted with age. The aggregate biomarker devised using random forests presented an AUC of 90.2%. However, the use of high-dimensional imaging data lowers the efficacy of systems.

On the contrary, Zhang et al. [9] attempted to predict future clinical change in a biomarker leveraging MRI and PET data and identified MCIp with an AUC of 76.8% using multikernel SVM. Other attempts of predicting longitudinal trajectories of biomarkers have also been recently reported [49]. Spasov et al. [46] applied the deep learning approach to MRI and clinical data to perform the said classification with an AUC of 92.5%.

While most studies focus on combining multimodal data and projecting them into a single space, we believe that considering predictors independently can preserve more information required for MCIp identification. Owing to the “lag” between brain atrophy and cognitive decline, when discriminating predictors are given independently to the classifier, enhanced classification performance is obtained as recorded. The method proposed in this paper has recorded the highest classification AUC of 95.7% predominantly because of involving the forecasted marker value at the time of conversion as a classification feature. Our approach is flexible and can be extended to incorporate other longitudinal predictors very easily.

## 5. Conclusion

In the current study, we presented a pipeline to identify the MCI patients who will develop AD in the future. Cognitive status and MRI measures were separately and collectively employed. The longitudinal trends observed in the predictors are utilized to forecast the future variability in the predictor values. The time-series data of markers are used to classify a subject as a progressor or a stable feature. Our best bimodal model forecasted the future values with a high precision (MAE: 3.64%) and yielded an excellent classification performance (AUC: 91.2%, accuracy: 84%) for 6-month-ahead conversion prediction. More stable results were obtained for 1-year-ahead AD diagnosis (MAE: 3.18%, AUC: 95.7%, accuracy: 81%). We conclude that it will be interesting to incorporate other predictors like PET and CSF measures in this framework and repeat the process for longer-term-ahead prediction. The use of a genetic algorithm or neural network for weight learning is also an explorable avenue in this domain to improve the results.

## Figures and Tables

**Table 1:** Previously performed longitudinal studies.

Author	Follow-up interval (months)	Follow-up duration (months)	Predictors	Dataset (MCIp/MCIs)	Accuracy (%)	AUC (%)
Guo [25]	6	36	MRI	43/35	84.6	92.5
Teng [26]	6	48	PET, NM	33/46	88.6	93.1
Lee [27]	6	24	MRI, CSF, NM		81.0	86.0
Huang [22]	6	48	MRI	70/61	79.4	81.2
Lee [23]	12	24	MRI	78/54	84.0 (female)	—
Arco [28]	6	12	MRI, NM	73/61	74.0	79.3
Runtti [24]	24	24	MRI, NM, CSF, genetic	126/174	76.9	82.3
Zhang [9]	6	24	MRI, NM, PET	35/50	78.4	76.8
Davatzikos [17]	12	36	MRI, CSF	69/170	55.8	73.4
Misra [8]	8	15	MRI	27/76	—	77.0

NM : neuropsychological measures, PET : positron emission tomography, MRI : magnetic resonance imaging, and CSF : cerebrospinal fluid.

**Table 2:** Subject demographic information.

		MCIp (*n* = 49)	MCIs (*n* = 70)

6 months	Age	73.3 ± 7.4	74.7 ± 7.3
	Gender (M/F)	29/20	36/34
	Education	15.8 ± 2.9	15.3 ± 2.9

		MCIp (*n* = 35)	MCIs (*n* = 50)

1 year	Age	73.5 ± 7.2	74.8 ± 7.3
	Gender (M/F)	2/15	29/21
	Education	16.0 ± 2.7	15.8 ± 2.7

**Table 3:** Neuropsychological measures used in this study (*M* ± SD: mean ± standard deviation).

Neuropsychological measure	MCIp	MCIs	*p* values
ADAS-cog total 11	19.9 ± 3.83	14.4 ± 5.22	<0.001
RAVLT	6.26 ± 1.71	9.13 ± 2.75	<0.001
CLOCKSCOR	4.12 ± 1.12	4.49 ± 0.64	0.069
COPYSCOR	4.76 ± 0.49	4.68 ± 0.65	0.463
LIMMTOT	7.00 ± 2.87	8.19 ± 2.82	<0.05
MMSE	26.82 ± 1.64	27.80 ± 1.52	<0.05
TRAASCOR	49.58 ± 28.11	37.01 ± 9.96	<0.05
TRABSCOR	125.6 ± 62	104.4 ± 49.4	0.061

**Table 4:** Top ranked markers and their *p*  values.

	6 months		1 year	
Rank	Marker	*p* values	Marker	*p* values
1	RAVLT	8.6 × 10^−12^	RAVLT	4.45 × 10^−9^
2	ADAS	2.1 × 10^−10^	ADAS	2.6 × 10^−8^
3	Volume of right amygdala	9.1 × 10^−07^	Cortical thickness of left isthmus cingulate	8.3 × 10^−6^
4	Volume of right entorhinal	1.7 × 10^−06^	Cortical thickness average of left fusiform	1.78 × 10^−5^
5	Volume of left amygdala.	2.0 × 10^−06^	Cortical thickness average of right isthmus cingulate	2.7 × 10^−05^

**Table 5:** Comparison of ground truth (GT) AUC and obtained AUC using the proposed method.

	6 months			1 year		
	Size of marker set	GT AUC (*p* value)	Proposed AUC (*p* value)	Size of marker set	GT AUC (*p* value)	Proposed AUC (*p* value)
NM	2	90.7% (1.7 × 10^−5^)	87.1% (1.9 × 10^−5^)\	4	93.7% (4.1 × 10^−6^)	91.5% (5.1 × 10^−6^)
MRI	13	88.71% (4 × 10^−6^)	88% (1.3 × 10^−6^)	13	91.4% (8.7 × 10^−7^)	90.5% (1.02 × 10^−6^)
NM + MRI	19	94.2% (4.8 × 10^−6^)	91.2% (4.6 × 10^−6^)	9	98% (8.5 × 10^−8^)	95.7% (4.05 × 10^−7^)

**Table 6:** Average MCIp vs. MCIs performance metrics.

	6 months ahead			1 year ahead		
	Accuracy (%) (*p* value)	Sensitivity (%)	Specificity (%)	Accuracy (%) (*p* value)	Sensitivity (%)	Specificity (%)
NM	77.0 (9 × 10^−5^)	73.5	80	84.7 (9.3 × 10^−6^)	82.9	86
MRI	79.8 (1.2 × 10^−5^)	71.8	71.8	83.5 (1.69 × 10^−5^)	74.28	90
NM + MRI	84.9 (3.9 × 10^−5^)	81.7	87.1	81 (1.43 × 10^−5^)	85.7	70

**Table 7:** Comparison with state-of-the-art results based on the ADNI dataset.

Author	Predictors	Dataset size	Validation	AUC (%)
Misra [8]	MR images	27 MCIp, 76 MCIs	LOO	77
Davatzikos [17]	MR images	69 MCIp, 170 MCIs	5-fold	73.4
Hinrichs [45]	MRI	48 AD, 66 CN, 119 MCI	LOO	79
Zhang [9]	MR images	38 MCIp, 50 MCIs	10-fold	76.8
Moradi [11]	MRI, NM, age	164 MCIp, 100 MCIs	10-fold	90
Arco [30]	MRI, NM	73 MCIp, 61 MCIs	LOO	79.23
Spasov [46]	MRI, NM	16 MCIp, 16 MCIs	10-fold	92.5
Guo [47]	MRI	43 MCIp, 35 MCIs	LOO	92.31
Platero [48]	MRI, NM	206 MCIp, 215 MCIs	10-fold	85.5
Proposed	MRI, NM	35 MCIp, 50 MCIs	5-fold	95.7

## Data Availability

In this paper, we have used data provided by Alzheimer's Disease Neuroimaging Initiative (ADNI). ADNI data are disseminated by the Laboratory for Neuro Imaging at the University of Southern California.

## Supplementary Materials

Supplementary Materials In ADNI, every subject is identified with a unique Roster ID (RID). The RIDs of the subjects considered in this study as MCIp and MCIs are available as supplementary file S1.
