# Supplementary material for: Early MCI-to-AD Conversion Prediction Using Future Value Forecasting of Multimodal Features
Source: Comput Intell Neurosci. 2021 Sep 24;2021:6628036. doi: 10.1155/2021/6628036 (PMC8487363; doi:10.1155/2021/6628036)
Supplement: Supplementary Materials — In ADNI, every subject is identified with a unique Roster ID (RID). The RIDs of the subjects considered in this study as MCIp and MCIs are available as supplementary file S1. [file 6628036.f1.docx]

**SUPPLEMETARY FILE: S1**

| **RID For Progressors** | **RID For Stables** |
| --- | --- |
| 57 | 107 |
| 108 | 116 |
| 126 | 150 |
| 187 | 158 |
| 214 | 169 |
| 231 | 200 |
| 294 | 273 |
| 314 | 285 |
| 331 | 290 |
| 362 | 291 |
| 388 | 307 |
| 390 | 324 |
| 513 | 351 |
| 649 | 448 |
| 675 | 464 |
| 702 | 481 |
| 708 | 546 |
| 725 | 557 |
| 835 | 621 |
| 839 | 626 |
| 906 | 644 |
| 952 | 656 |
| 978 | 673 |
| 1007 | 679 |
| 1010 | 698 |
| 1057 | 746 |
| 1121 | 771 |
| 1135 | 800 |
| 1240 | 912 |
| 1299 | 919 |
| 1315 | 1045 |
| 1326 | 1074 |
| 1351 | 1118 |
| 1389 | 1122 |
| 1425 | 1140 |
|  | 1155 |
|  | 1182 |
|  | 1186 |
|  | 1187 |
|  | 1227 |
|  | 1246 |
|  | 1260 |
|  | 1268 |
|  | 1269 |
|  | 1300 |
|  | 1314 |
|  | 1384 |
|  | 1414 |
|  | 1418 |
|  | 1419 |
